# The Relationship Between People’s Environmental Considerations and Pro-environmental Behavior in Lithuania

**DOI:** 10.3389/fpsyg.2019.02319

**Published:** 2019-10-15

**Authors:** Audra Balundė, Goda Perlaviciute, Linda Steg

**Affiliations:** ^1^Environmental Psychology Research Centre, Institute of Psychology, Mykolas Romeris University, Vilnius, Lithuania; ^2^Department of Psychology, Faculty of Behavioural and Social Sciences, Groningen University, Groningen, Netherlands

**Keywords:** pro-environmental behavior, non-WEIRD country, biospheric values, environmental self-identity, environmental considerations

## Abstract

Given the need for global action on climate change, it is crucial to comprehend which factors motivate people in different countries to act more pro-environmentally. Lithuania is a post-socialist country that has recently increased commitment to foster pro-environmental behavior of individuals, by implementing interventions that target mainly the personal costs and benefits of relevant behaviors. Yet, research suggests that people’s general environmental considerations, namely biospheric values and environmental self-identity, can drive people’ pro-environmental behavior and may be important targets for interventions. These studies, however, have been mostly conducted in Western Europe and the United States, with limited evidence of relationship between people’s biospheric values, environmental self-identity and pro-environmental behaviors across different countries and cultures. We performed a correlational study with a convenience sample in Lithuania (*n* = 334). Consistent with previous studies and the theory, our study revealed that people’s general environmental considerations were positively related with recycling and environmental activism, but not with fuel-efficient driving and the use of sustainable transportation in Lithuania. We conclude that general environmental considerations are related to pro-environmental behaviors beyond Western Europe and the United States. Yet, future studies need to examine the boundary conditions of this relationship and test whether interventions targeting environmental consideration can be effective to promote pro-environmental behavior.

## Introduction

Anthropogenic causes of environmental problems such as climate change have been widely acknowledged (Stern et al., 2016; Intergovernmental Panel on Climate Change [IPCC], 2018). Environmental problems could therefore be reduced if people acted more pro-environmentally. Many countries have already committed to take measures to reduce climate change (European Commission, 2015), yet the efficiency of implementation and scale of these measures differs across countries (Intergovernmental Panel on Climate Change [IPCC], 2018). Such measures will be more effective if they address key antecedents of pro-environmental actions of citizens of these different countries (Steg and Gifford, 2017). To this end, the question is which factors are related to pro-environmental behavior across different countries and cultures (Mancha and Yoder, 2015; Morren and Grinstein, 2016). Such knowledge is crucial for developing effective measures to promote pro-environmental behavior in countries across the world (Steg et al., 2014a; Intergovernmental Panel on Climate Change [IPCC], 2018).

Research shows that people’s environmental considerations, such as biospheric values and environmental self-identity, are related to pro-environmental behavior. Pro-environmental behavior can be defined as all possible actions aimed at avoiding harm to and/or safeguarding the environment (Steg and Vlek, 2009), either performed in public (e.g., participation in environmental movements) or private domains (e.g., recycling; Hadler and Haller, 2011). Values have been found to be an important antecedent of a variety of pro-environmental behaviors (Nordlund and Garvill, 2002; Abrahamse and Steg, 2013; Steg et al., 2014a, 2015). Four types of values have been shown to be particularly important in explaining pro-environmental behavior, namely biospheric values (caring about nature and environment protection), altruistic values (focusing on the well-being of others), egoistic values (safeguarding and promoting personal resources), and hedonic values (focusing on seeking pleasure and reducing effort) (Steg and De Groot, 2012; Steg et al., 2014b). Pro-environmental behaviors often imply personal costs, while the benefits are mostly for the environment and society at large. This explains why, compared to other values, particularly people’s biospheric values are positively and strongly related to pro-environmental behavior (Nordlund and Garvill, 2002; Steg et al., 2011, 2014a). Biospheric values motivate people to act pro-environmentally, even when the behavior is somewhat costly (Verplanken and Holland, 2002; Steg et al., 2014a; Loebnitz and Aschemann-Witzel, 2016; Steg, 2016). For example, the more individuals endorse their biospheric values, the more they recycle and drive in an energy-efficient manner (van der Werff et al., 2014b), engage in environmental activism (Stern et al., 1999; Steg et al., 2011), eat less meat and take shorter showers (Thøgersen and Ölander, 2002; Steg et al., 2014b), intend to reduce car use and accept car use reduction policies (De Groot and Steg, 2007), accept energy policies aimed at reducing household CO_2_ emissions (Steg et al., 2011), and adopt renewable energy systems at home (van der Werff and Steg, 2016).

Biospheric values represent general goal to protect the environment, which is why they are related to many different pro-environmental behaviors. Yet, it also implies that they are related to behaviors mostly indirectly (Steg et al., 2014a; Dietz, 2015). An important factor that could explain the relationship between biospheric values and pro-environmental behavior is environmental self-identity (van der Werff et al., 2013b). Environmental self-identity reflects the degree to which a person thinks of himself or herself as an individual who acts in environmentally friendly manner (van der Werff et al., 2013a). People are motivated to act in line with how they see themselves in order to be or appear to be consistent (Bem, 1972). The stronger their environmental self-identity, the more individuals tend to recycle (Mannetti et al., 2004; Whitmarsh and O’Neill, 2010), use sustainable transportation and drive fuel-efficiently (van der Werff et al., 2013b), engage in environmental activism (Fielding et al., 2008), intend to consume less meat (van der Werff et al., 2013b), and have a preference for environmentally friendly products (van der Werff et al., 2014b; Barbarossa and De Pelsmacker, 2016).

**FIGURE 1:**
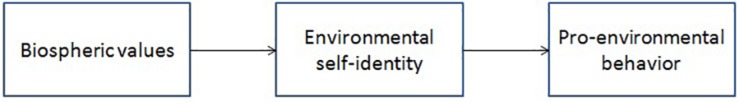
Theoretical model on the relationship between biospheric values, environmental self-identity and pro-environmental behavior (adapted from van der Werff et al., 2014b).

So far, biospheric values and environmental self-identity have mostly been studied separately. Yet, building on the above, a theoretical model has been introduced, which postulates that environmental self-identity mediates the relationship between biospheric values and pro-environmental behaviors (Figure 1; van der Werff et al., 2014b). A few studies have tested the full model of relationship between biospheric values, environmental self-identity and pro-environmental behavior, providing initial empirical support for the model, including evidence for the proposed mediation effect, via correlational as well as experimental designs (van der Werff et al., 2013b, 2014b; Gatersleben et al., 2014; van der Werff and Steg, 2016).

Importantly, these studies have been carried out exclusively in Western European countries with relatively long history of policies and measures aimed at promoting individual pro-environmental behavior. This raises a question whether similar relationship between these key variables can be found in other countries, which are not so-called WEIRD countries (Western, Educated, Industrialized, Rich and Developed; Henrich et al., 2010) and which are only starting to develop and implement policies and measures to promote individual pro-environmental behavior.

Some initial evidence from non-Western countries supports *parts* of the relationships proposed in the theoretical model (Figure 1). For example, in Latin America, notably Argentina, biospheric values were related to household energy savings (Jakovcevic and Reyna, 2016), and intention to reduce passenger car use and acceptability of passenger car taxation policies (Jakovcevic and Steg, 2013). In addition, biospheric values were related to energy conservation behavior in Hungary (De Groot et al., 2012) and Turkey (Sahin, 2013), and acceptability of car use reduction policies in Japan and Russia (Hiratsuka et al., 2018; Ünal et al., 2019). These studies however did not include environmental self-identity. One study found that identifying oneself as an environmentally friendly consumer mediated the effects of biospheric values on intentions to buy energy-efficient appliances in Vietnam (Nguyen et al., 2016). However, seeing oneself as an environmentally friendly consumer is a very specific measure that is less likely to be related to a variety of pro-environmental behaviors than a more general measure of environmental self-identity. To conclude, the proposed relationship in the literature between biospheric values, environmental self-identity, and different pro-environmental behaviors (Figure 1; van der Werff et al., 2014b) has not been studied outside affluent Western Europe; the current study addresses this gap in the literature by testing above mentioned relationship in different country, namely Lithuania.

Lithuania is a post-socialist country and relatively young member of the European Union (EU) that differs in many respects from the Western EU and from countries where previous studies on the relationship between biospheric values, environmental self-identity and environmental behaviors have been conducted. After gaining its independence in 1990, Lithuania went through radical changes of economic system: from state-run to market-oriented. It also encountered challenges while reestablishing its national identity. Since joining the European Union and NATO in 2004 Lithuania demonstrated a quick development and growth of economy. Being a part of these internationally acknowledged alliances’ including OECD indicates that Lithuania’s economic performance approaches that of developed countries. Yet, despite economical advancements, Lithuania encounters issues that are not common to Western societies, especially the so-called WEIRD countries (Henrich et al., 2010). These issues include high rates of people living at risk of poverty and social exclusion (Eurostat, 2017b), one of the lowest income among EU countries (Eurostat, 2019), large development gaps between large cities and areas beyond them (Pociūtė-Sereikienė, 2019), high rates of unemployment in rural areas, high emigration rates and drain of the intellectual capital that flows to Western countries (Ubarevičienė and Van Ham, 2017). What is even more relevant in the context of the current study, is that Lithuanians do not recognize climate change as important, particularly compared to citizens of affluent countries such as Sweden, Finland, Denmark, Ireland, Belgium, France and the Netherlands (Eurobarometer, 2018). Furthermore, unlike in Western European countries, the so-called post-materialist values (Inglehart, 2018) are not dominant in Lithuanian society; on the contrary, with the slight fluctuations, materialist values became increasingly more expressed from 1990 to 2008 (Savicka, 2016), during the period of economic growth. Although the latest data shows consistent slow decrease in materialist values and increase of post-materialist values in Lithuania, materialist values remain dominant (Inglehart, 2018). Materialistic values can indeed inhibit pro-environmental actions (Wang et al., 2019), therefore, the question is whether there is a relationship between environmental considerations and pro-environmental behavior in Lithuania.

One could speculate that in a country with such prominent other issues besides the environment, environmental considerations are not strongly related to concrete pro-environmental behaviors. If anything, Lithuanians may link pro-environmental behaviors primarily to immediate practical concerns, such as costs and convenience. This assumption seems to be entrenched in policies and measures aimed at promoting pro-environmental behaviors in Lithuania. These measures have mostly aimed at making pro-environmental behavior beneficial and making the environmentally harmful behavior costly, thus focusing exclusively on reducing individual costs and increasing individual benefits of pro-environmental behavior. Examples include infrastructure developments and technological improvements (e.g., building cycling paths, improving public transportation facilities, improving and establishing new recycling facilities, and implementing cleaner technologies in industry, among others; European Environment Agency [EEA], 2015) as well as financial incentives (e.g., deposits for recycled bottles and cans, taxes for commercial motor vehicles, and a free parking service for users of electric vehicles; Legislative Collection [LC], 2014b; No. 5579). Notably, such measures typically do not address general environmental considerations to motivate people to engage in the relevant actions. While there have been some informational campaigns that target people’s awareness of environmental problems (Legislative Collection [LC], 2014a; No. 2884), such campaigns typically do not inform people about which individual actions could contribute to resolving these issues. Alternatively, one could also expect that biospheric values and environmental self-identity are universal general factors that are related to environmental behavior across countries and cultures, therefore environmental considerations will be related to environmental behavior in Lithuania. Since both cases are likely, it is very relevant to study whether and to what extent the relationship between biospheric values, environmental self-identity, and pro-environmental behaviors exists in Lithuania.

We study the relationship between biospheric values, environmental self-identity, and several pro-environmental behaviors, which we consider highly relevant in Lithuania, namely recycling, environmental activism, and transportation behaviors, specifically fuel-efficient driving and the use of sustainable transportation. Notably, although the amount of recycled waste in Lithuania increased from 2% in 2004 to 30% in 2014, probably largely due to improved recycling facilities, it remains below the EU target to recycle 50% of waste in 2020 (European Environment Agency [EEA], 2017). Next, Lithuania is behind most of the EU countries in the use of public transportation, with 89.2% of citizens commuting by car (Eurostat, 2017a). This is noteworthy, since 25.4% of greenhouse gas emissions in Lithuania are caused by the transportation sector (Lithuania Ministry of Environment [LME] and Environmental Protection Agency [EPA], 2017). Finally, environmental activism is interesting because it had favorable conditions to occur in Lithuania. In 2016 (when this study was conducted) a critical mass of NGOs’ was formed in Lithuania. These NGO’s aim to educate society about environmental issues and promote citizens’ environmental activism. This has been done mainly through informational campaigns [e.g., raising awareness on environmental issues such as climate change via public service announcements (PSAs’)], educational campaigns (e.g., “Green Olympics”) and public events (e.g., creative recreational activities such as festivals in nature in protected areas) (Kriauciunaite and Telesiene, 2009). Although fragmentally and mostly on a small scale, NGOs’ have therefore been targeting people’s environmental considerations to promote pro-environmental behavior, and environmental activism in particular. Yet, the question remains whether or not environmental considerations are related to pro-environmental behaviors in Lithuania, which represents post-socialist countries that are only starting to promote pro-environmental behavior.

## Materials and Methods

### Participants and Procedure

The data was collected in April–July 2016 in Lithuania, using a convenience sampling method. Most of the participants completed online questionnaires disseminated through social networks Facebook and Google+, additionally asking the participants to forward the link to the questionnaire to as many people as possible. In total 266 people completed the questionnaire (79.6% of the total sample; the response rate is not known as we did not track to how many contacts the participants forwarded the questionnaire). Besides, we approached students at a local university during psychology lectures and asked them to complete a paper version of the questionnaire (8.1% of the sample; *n* = 27; response rate: 90%). The rest of the participants were recruited by asking acquaintances of the first author to fill in a paper version of the questionnaire (12.3% of the sample; *n* = 41; response rate: 41%). Participants’ confidentiality and possibility to withdraw from the study at any stage were assured.

First, participants’ values were measured and afterward their pro-environmental behaviors and environmental self-identity.^1^ Additionally, questions about demographic characteristics were included at the end of the questionnaire. Eight participants did not complete a substantial part of the questionnaire and their data were excluded from the analysis, therefore the final sample consisted of 334 responses. In total 65 men and 266 women participated in the study; three participants did not specify their gender. The age of participants ranged from 18 to 73 (M_age_ = 34.28, SD_age_ = 12.28). In total 76.3% of the participants had obtained higher education, 22.8% had vocational or lower education, and 0.9% did not specify their education level. Income distribution was as follows: 21.9% had a monthly personal income of ≤300 €, 36.5% had a monthly personal income of ≤600 €, 17.1% had a monthly personal income of ≤800 €, 6.6% had a monthly personal income of ≤1000 €, 15.2% had a monthly personal income of >1000 €, and 3.3% participants did not specify their income.

*A priori* power analysis (Soper, 2006–2017) revealed that *n* = 161 is the minimum and *n* = 227 is the recommended sample size to conduct a valid SEM analysis to test the model (when effect size is 0.30, statistical power is 0.80, level of significance is 0.05, with six latent variables and 17 observed variables). The sample size of this study, *n* = 334, was therefore sufficient.

### Measures

We used established instruments to measure the key variables of interest, which we translated into Lithuanian. Translation and back-translation procedures were performed.^2^ For all measures that consisted of three or more items, we used Cronbach’s alpha to assess scale reliability. For measurements consisting of two items, we calculated correlation coefficients. All scales included in the study were reliable.

#### Values

A short version of the Schwartz’s values scale (Schwartz, 1992, 1994) developed by De Groot and Steg (2007) was used to measure people’s values. Instrument was tested and validated in numerous studies (e.g., Steg et al., 2014b). Participants indicated on a nine-point scale to what extent they find different values important as guiding principles of their life. A scale ranged from –1 opposed to my guiding principles, 0 not important, to 7 of supreme importance. The scale included four items that measure biospheric values (e.g., “Respecting the earth: harmony with other species”; α = 0.89). Higher scores mean stronger endorsement of biospheric values.

We used the Oblique Multiple Group (OMG) method to test whether the data supports the *a priori* assignment of the value items to the four value dimensions (Stuive et al., 2008), a method that is commonly used to test whether the four types of values can be distinguished empirically (e.g., Steg et al., 2014b). The analysis confirmed the distinction of biospheric values from altruistic, egoistic, and hedonic values. Notably, the items that are theoretically meant to measure biospheric values indeed correlated stronger with the biospheric values scale than with the other value scales, when controlled for self-correlations (Table 1).^3^

**TABLE 1:** Correlations between the value items and the corresponding value scales.

**Scale**	**Biospheric**	**Altruistic**	**Egoistic**	**Hedonic**
	**values**	**values**	**values**	**values**
**Biospheric values**				
Respecting the earth	**0.663**	0.372	–0.017	0.054
Unity with nature	**0.647**	0.279	0.055	0.076
Protecting the environment	**0.726**	0.361	0.097	0.167
Preventing pollution	**0.710**	0.357	0.059	0.103
**Altruistic values**				
Equality	0.276	**0**.**280**	0.093	0.204
A world at peace	**0.442**	0.239	0.089	0.211
Social justice	0.355	**0**.**360**	0.116	0.092
Helpful	0.297	**0**.**329**	0.175	0.045
**Egoistic values**				
Social power	–0.012	0.074	**0**.**383**	0.242
Wealth	–0.028	0.016	0.314	**0**.**487**
Authority	–0.052	0.013	**0**.**444**	0.348
Influential	0.103	0.180	**0**.**358**	0.282
Ambitious	0.233	**0**.**307**	0.206	0.259
**Hedonic values**				
Pleasure	0.124	0.135	0.327	**0**.**586**
Enjoying life	0.012	0.091	0.313	**0**.**532**
Self-indulgent	0.164	0.188	0.330	**0**.**590**

*Correlation coefficients are corrected for self-correlation and test-length. The highest correlation coefficients of each item, are marked in bold. The tested item grouping explains 62.43% of the observed variance.*

#### Pro-environmental Behavior

To measure pro-environmental behaviors, we used items from the General Ecological Behavior instrument (Kaiser and Wilson, 2004). Participants rated the frequency of their engagement in each of the behaviors on a Likert type scale, varying from 1 never to 5 very often. Two items measured recycling behavior: “I collect and recycle used paper” and “I bring empty bottles to a recycling bin” (*r* = 0.46, *p* < 0.01); three items measured environmental activism: “I boycott companies with an unecological background,” “I read about environmental issues,” and “I talk with my friends about environmental pollution, climate change, and/or energy consumption” (α = 0.75); two items measured fuel-efficient driving: “I keep the engine running while waiting in front of a railroad crossing or in a traffic jam” and “At red traffic lights, I keep the engine running” (scores were reverse-coded; higher scores mean more fuel-efficient driving; *r* = 0.49, *p* < 0.001); and three items measured the use of sustainable transportation modes: “I ride a bicycle or take public transportation to work or school,” “I drive my car to the city” (reverse coded), and “In nearby areas (around 30 km), I use public transportation or ride a bike” (α = 0.87).

#### Environmental Self-Identity

A validated instrument was used to measure individuals’ environmental self-identity (van der Werff et al., 2013a, b). Participants indicated on a Likert type scale, varying from 1 totally disagree to 7 totally agree to what extent they consider themselves as individuals who act in an environmentally friendly way (e.g., “Acting pro-environmentally in an important part of who I am”; α = 0.81). Higher scores reflect a stronger environmental self-identity.

## Results

### Data Analysis Strategy

The statistical software SPSS 23.0 and Mplus 7.4 (Muthén and Muthén, (1998–2010)) were used for the data analyses. To test the relationship between key variables we followed statistical procedures that are commonly applied to test the theoretical model (van der Werff et al., 2013b, 2014b; Gatersleben et al., 2014; van der Werff and Steg, 2016). We first studied bivariate correlations to examine the relationships between the key variables. Next, to examine the relationship between the environmental considerations and the four pro-environmental behaviors as proposed by the theoretical model (Figure 1), we used Structural Equation Modeling (SEM) with a maximum likelihood estimator (ML) that, treats biospheric values and environmental self-identity as predictors of the four types of pro-environmental behaviors, and environmental self-identity as a mediating variable in the relationship between biospheric values and the different behaviors.^4^

### Relationship Between People’s Biospheric Values, Environmental Self-Identity and Pro-environmental Behavior

Bivariate correlations between all variables measured in this study (as well as means and standard deviations) are provided in Table 2. We further discuss specifically the relationships that are relevant for the current research questions. Biospheric values and environmental self-identity correlated positively with all pro-environmental behaviors, except for the use of sustainable transportation modes. Biospheric values and environmental self-identity were also correlated positively and strongly.^5^

**TABLE 2:** Bivariate correlations between values, environmental self-identity, pro-environmental behaviors and demographic characteristics.

	**M/(SD)**	**1**	**2**	**3**	**4**	**5**	**6**	**7**	**8**	**9**	**10**	**11**	**12**
1. Biospheric values	5.58(1.34)	–											
2. Altruistic values	5.60(1.08)	0.55^∗∗∗^	–										
3. Hedonic values	5.05(1.52)	0.13^∗^	0.23^∗∗∗^	–									
4. Egoistic values	3.13(1.59)	0.003	0.14^∗^	0.52^∗∗∗^	–								
5. Environmental self-identity	5.25(1.12)	0.62^∗∗∗^	0.42^∗∗∗^	–0.05	–0.01	–							
6. Recycling	3.90(1.12)	0.23^∗∗∗^	0.08	–0.19^∗∗∗^	–0.10	0.33^∗∗∗^	–						
7. Environmental activism	2.48(0.92)	0.43^∗∗∗^	0.23^∗∗∗^	−0.11^∗^	−0.12^∗^	0.47^∗∗∗^	0.35^∗∗∗^	–					
8. Sustainable transportation use	3.00(1.40)	–0.04	–0.02	–0.08	−0.14^∗^	–0.02	0.07	0.01	–				
9. Fuel-efficient driving	2.25(1.16)	0.18^∗∗^	0.002	–0.06	–0.04	0.18^∗∗^	0.06	0.22^∗∗∗^	0.26^∗∗∗^	–			
10. Gender		0.05	0.09	–0.02	–0.05	0.13^∗^	0.14^∗^	0.13^∗^	0.08	–0.10	–		
11. Age	34.29(12.29)	0.29^∗∗∗^	0.10	–0.18^∗∗^	0.01	0.38^∗∗∗^	0.28^∗∗∗^	0.32^∗∗∗^	–0.18^∗∗^	0.19^∗∗^	0.02	–	
12. Education		0.07	–0.02	–0.09	0.02	0.13^∗^	0.17^∗∗^	0.20^∗∗∗^	–0.10	–0.08	0.03	0.41^∗∗∗^	–
13. Income		0.09	–0.04	0.03	0.07	0.06	0.08	0.03	–0.21^∗∗∗^	–0.03	–0.17^∗∗^	0.17^∗∗^	0.29^∗∗∗^

*^∗^*p* < 0.05, ^∗∗^*p* < 0.01, ^∗∗∗^*p* < 0.001.*

The model^6^ of the relationship between environmental considerations and pro-environmental behaviors (Figure 1) fitted the data sufficiently well (χ^2^ = 217.72, *p* < 0.01, df = 104, CFI = 0.95, TLI = 0.94, RMSEA [90% CI] = 0.06 [0.05;0.07], SRMR = 0.05) and could be considered as acceptable (Little, 2013).^7^ The results are illustrated in Table 3.

**TABLE 3:** Relationship between biospheric values and pro-environmental behaviors, mediated by environmental self-identity.

**Indirect and direct paths**	**Estimate [95% BC CI]**	**SE**
Indirect effect of BIOS on REC via ESI	0.34^∗∗^[0.12;0.55]	0.11
Direct effect of BIOS on REC	−0.01[−0.26;25]	0.13
Indirect effect of BIOS on EA via ESI	0.29^∗∗^[0.08;0.50]	0.11
Direct effect of BIOS on EA	0.22[−0.01;0.45]	0.12
Indirect effect of BIOS on FED via ESI	0.16[−0.04;35]	0.10
Direct effect of BIOS on FED	0.06[−0.21;0.34]	0.14
Indirect effect of BIOS on STM via ESI	0.03[−0.12;0.19]	0.08
Direct effect of BIOS on STM	−0.10[−0.31;0.12]	0.11

*Estimate – standardized estimates; SE – standard error; BC CI – bias-corrected confidence intervals. BIOS – biospheric values; ESI – environmental self-identity; REC – recycling; EA – environmental activism; FED – fuel-efficient driving; STM – the use of sustainable transportation modes. ^∗∗^*p* < 0.01.*

In line with the theoretical model, we found that biospheric values were related to environmental self-identity (β = 0.69, *p* < 0.01). Biospheric values had no direct positive relationship with the studied behaviors when environmental self-identity was included as a mediator. We found significant indirect relationship between biospheric values and environmental activism as well as recycling via environmental self-identity, but no relationship was found between biospheric values and fuel-efficient driving and the use of sustainable transportation modes via environmental self-identity (Table 3).

Environmental self-identity explained 23% of variance in recycling behavior, while biospheric values, when including environmental self-identity as a mediator, explained 10% of variance in recycling. Environmental self-identity explained 35% of variance in environmental activism, while biospheric values, when including environmental self-identity as a mediator, explained 17% of variance in environmental activism.

## Discussion

Given the global urgency to combat climate change, it is highly relevant to investigate general factors that relate to various pro-environmental behavior across different countries and cultures. This is particularly important for countries where interventions to promote pro-environmental behavior are only starting to enter political agendas and where there has been little research into factors related to pro-environmental behaviors. We studied the relationship between general environmental considerations, namely biospheric values and environmental self-identity, and pro-environmental behaviors in Lithuania, a post-socialist and non-WEIRD country.

We found that biospheric values and environmental self-identity were strongly correlated with each other and related to some types of pro-environmental behaviors in Lithuania, namely recycling and environmental activism. This provides further support for the relationship between these key variables, as theorized and initially supported in past studies (van der Werff et al., 2013b, 2014b; Gatersleben et al., 2014; van der Werff and Steg, 2016), and gives first evidence that this relationship can hold in countries outside affluent Western-Europe. Together with studies that have tested parts of this theoretical model in other parts of the world, the current findings give evidence that also in countries outside Western-Europe and the United States, stronger general environmental considerations are associated with concrete pro-environmental behaviors (De Groot et al., 2012; Jakovcevic and Steg, 2013; Sahin, 2013; Jakovcevic and Reyna, 2016; Nguyen et al., 2016; Hiratsuka et al., 2018; Ünal et al., 2019).

While biospheric values and environmental self-identity were positively related with recycling and environmental activism, both variables were not significantly related with fuel-efficient driving and the use of sustainable transportation modes. This could be due to various reasons. First, using sustainable transportation modes may be perceived as a personally costly behavior in Lithuania, and it seems that environmental considerations are poorer predictors of relatively costly behaviors (Whitmarsh and O’Neill, 2010; Gatersleben et al., 2014). Using sustainable transportation modes in Lithuania can be inconvenient and time-consuming, for example because of outdated vehicles (e.g., buses) and absence of conditioning/heating systems in public transport. Cycling infrastructure in Lithuania is still underdeveloped, which makes cycling not easy and often even not safe. The relatively high personal costs of sustainable transportation behaviors in Lithuania could prevent people from engaging in these behaviors, even if they have strong biospheric values and a strong environmental self-identity. Future studies could examine whether and how perceived costs, as well as perceived benefits, of different pro-environmental behaviors affect the relationship between people’s environmental considerations and engagement in these behaviors. Second, especially for fuel-efficient driving, such as stopping the car engine when waiting at a traffic light, people may have never considered the environmental impact of such behavior, partly because such behaviors are never presented in interventions as having impact on the environment. Hence people may not take their environmental considerations into account in such behavioral choices. Future studies need to test the degree to which people are aware of the environmental impact of different behaviors and how that affects the relationship between their environmental considerations and engagement in these behaviors.

Building on the current findings, the next critical step is to test the effects of interventions that target people’s environmental considerations on pro-environmental behaviors. This is especially important in less affluent countries with limited budgets for environment interventions and in countries where practices fostering pro-environmental behavior are just starting. These countries are in need of evidence-based knowledge that could inform effective and efficient policies and measures to meet the climate goals. Our study gives initial evidence that general environmental considerations are related with specific pro-environmental behaviors in Lithuania, which is an important starting point to develop and test interventions that target environmental considerations. While interventions that make pro-environmental behaviors less costly are important, because they reduce barriers to the behavior, it may also be important at the same time to target environmental considerations in such interventions, explicitly linking these interventions to environmental considerations (van der Werff et al., 2013a, b; van der Werff and Steg, 2016). For example, interventions could aim at strengthening individuals’ environmental self-identity (Fanghella et al., 2019) by reminding people of their pro-environmental behavior performed in the past, which has been found to reinforce their environmental self-identity, eventually resulting in other environmentally beneficial actions (van der Werff et al., 2014a). The effectiveness of such interventions could be tested via experimental and/or field studies, more specifically it could be tested which interventions are effective in strengthening environmental self-identity and whether they indeed result in more pro-environmental behavior.

A few concerns regarding this study should be considered when interpreting the results. We used a convenience sample, which included more females and higher levels of education and income compared to the general population in Lithuania. Yet, the goal of the current research was to test the relationship between people’s environmental considerations and pro-environmental behaviors. While the absolute values of these variables could potentially differ across different demographic groups, we do not have a reason to expect that this would change the relationship between these variables (see also Schultz et al., 2005; De Groot and Steg, 2007; De Groot et al., 2012; Bhushan et al., 2019). We indeed found no effects of gender on the relationship between people’s environmental considerations and pro-environmental behaviors, and the relationship remained the same irrespective of the method of the data collection. Furthermore, our sample is likely to be comparable to the general population in terms of exposure to and experience with interventions aimed at promoting pro-environmental behavior in Lithuania. Future studies could explore whether findings of this research could be replicable in a more representative sample.

Next, we used self-reported measures of environmental behavior. Future studies could examine whether similar results will be found when including measures of actual behaviors, for example by weighing recycled materials of households (Bartelings and Sterner, 1999), reaching out to individuals who actually are (not) members of environmental organizations and/or studying people’s voting behaviors and donations to environmental organizations (Alisat and Riemer, 2015), using driving simulator for measuring fuel-efficient driving (Dogan et al., 2011; Zhao et al., 2015), and employing GPS technologies to trace daily transportation patterns (Bolderdijk et al., 2011; Houston et al., 2014), among others.

Further, the relationship between biospheric values and environmental self-identity was rather strong that could lead one to concerns regarding their discriminant validity. Strong relationship between the two constructs can be expected based on theory (e.g., Schwartz, 1992; Verplanken and Holland, 2002; Hitlin, 2003; Bardi et al., 2014). At the same time, the two constructs have been distinguished on theoretical grounds (Whitmarsh and O’Neill, 2010; van der Werff et al., 2013b, 2014b), and studies conducted in six different samples confirmed that they can be empirically distinguished (van der Werff et al., 2013b, 2014b). Yet it is important for future research to test whether the current findings regarding this strong correlation can be replicated in Lithuania and if so whether the two constructs can be empirically distinguished in Lithuania as it was done in other countries (van der Werff et al., 2013b, 2014b). Future research is also needed to find out what could cause some overlap between the two constructs in Lithuania.

Also, the variables of the current study were distributed on the right side from the zero (except egoistic and hedonic values that are deviated to the right side to a lesser extent). As suggests literature such deviation could yield stronger correlations than they actually are (Podsakoff et al., 2003). Yet in the current study, we found significant relationship between the environmental considerations and pro-environmental behaviors, but not for all of them. This indicates that probably relationships were not affected by certain biases that strong, because some relationships were non-significant. Rather, this points out that there may be important reasons why environmental considerations relate to some behaviors and not to others (e.g., high behavioral costs). Future studies could test whether similar results can be found when questionnaire order effects are reduced, for example, by randomizing instruments’ items (Podsakoff et al., 2003) and measuring key variables in different time points (e.g., van der Werff et al., 2013b, 2014b).

Finally, based on the correlational study, we cannot draw conclusions about the causal relationship between people’s environmental considerations and pro-environmental behavior. Yet, there is initial evidence from prior research on the causal direction of the relationships for parts of the model. For example, a two-wave longitudinal study with a representative Danish sample concluded that stronger universalism values, which incorporated biospheric values (e.g., protecting the environment) and altruistic values (e.g., social justice), predicted pro-environmental behavior (Thøgersen and Ölander, 2002). Specifically, a cross-lagged analysis indicated that values in the first measurement wave predicted pro-environmental behavior in the second wave, while behavior in the first wave had no effects on values in the second wave (Thøgersen and Ölander, 2002). Further, experimentally varying the levels of environmental self-identity in an experiment caused changes in people’s choice for environmentally (un)friendly products (van der Werff et al., 2014b), which implies causality. Moreover, the causal relationship between values, identity and behavior have strong theoretical underpinnings (Schwartz, 1977; Stern, 2000). Yet, additional studies are still needed to establish causality in the full model of relationship between biospheric values, environmental self-identity and pro-environmental actions. Notably, the participants in our study first reported their pro-environmental behaviors and afterward environmental self-identity. It has been established that reminding individuals of their past pro-environmental behaviors could affect their environmental self-identity (van der Werff et al., 2014a, b). Yet, since we found different relationship between the environmental considerations and some pro-environmental behaviors, but not for other pro-environmental behaviors, such order effects are not likely to have impacted on our results. Future studies could test the order effects further by counterbalancing the order of the items.

All in all, we find that also in a post-socialist non-WEIRD country general environmental considerations, namely biospheric values and environmental self-identity, are related to some pro-environmental behaviors. This is an important finding since current policies and interventions targeting pro-environmental behavior in Lithuania seem to rely on the assumption that pro-environmental behaviors are related to immediate, practical considerations only. With the current findings, we hope to provide impetus for future research to test the effects of interventions targeting environmental considerations on pro-environmental behavior in countries that have been understudied so far, such as Lithuania.

## Data Availability Statement

The dataset analyzed for this study and related materials can be found in the Open Science Foundation repository https://osf.io/qn9fp/?view_only=055ffd0809454c0ba6bd2f2597097c08.

## Ethics Statement

This research was conducted in accordance with the recommendations of and approved by the Psychological Research Ethics Committee of the Mykolas Romeris University. Informed consent was obtained from study participants; it was considered that by filling in the survey participants gave their consent to participate. Anonymity of participants was assured. Study participants were informed that their participation is voluntary, and that they can refuse to participate or withdraw from the study at any stage. Participants were also informed about the aims of the study and that the data will be used for scientific purposes only. Participation in the online and paper surveys was anonymous; no data was collected that can be linked to the participants’ identity.

## Author Contributions

AB designed the study, collected and analyzed the data, and drafted the manuscript. GP considerably contributed to the writing all parts of the manuscript. LS made conceptual suggestions and critical revisions of the manuscript.

## Conflict of Interest

The authors declare that the research was conducted in the absence of any commercial or financial relationships that could be construed as a potential conflict of interest.

## Footnotes

1 The current research was part of a larger survey on pro-environmental behavior in Lithuania. We only discuss measures that are directly relevant to this study; other measures are available from the first author upon request.

2 First, a Lithuanian native speaker, who is an English linguist-translator, translated the original scales from English to Lithuanian. Afterward, a second English linguist-translator back-translated the Lithuanian version to English. Next, the first author and another researcher in Psychology (both native Lithuanian speakers) translated the English version into Lithuanian. Finally, the first author, the second researcher in Psychology, and an expert in Lithuanian language checked for inconsistencies between the different translated versions and made corrections where necessary.

3 The item measuring altruistic values, namely “A world at peace,” correlated more strongly with the biospheric values scale and two items measuring egoistic values, namely “Ambitious” and “Wealth” correlated more strongly with the altruistic and hedonic values scales, respectively (see Table 1). Given that we focus in this study on particularly biospheric values and that the items measuring biospheric values formed a reliable scale, we decided to comply with the theoretically and empirically established structure of the instrument. Interestingly, studies in some other post-socialist counties, namely Czechia (De Groot and Steg, 2007) and Hungary (De Groot et al., 2012), also found that “A world at peace” correlates most strongly with the biospheric values scale. This could indicate that this value represents more biospheric than altruistic values in post-socialist countries. The studies in these countries also found a relatively strong positive correlation between “Ambitious” and the altruistic values scale. Materialism values dominate in comparison to post-materialism values in these countries, as in Lithuania (Inglehart, 2018). People in these countries value materialistic well-being and hard work (Inglehart, 2018) and may therefore perceive being ambitious, hardworking and aspiring as key qualities for the well-being of the society. These explanations are highly speculative, however, and required further examination. The positive correlation between “Wealth” and the hedonic values scale is likely since both types of values belong to the same dimension of self-enhancement values (Schwartz, 1992, 1994).

4 Please note that because of the correlational nature of the data, we cannot draw firm conclusions on causal relationships. We thank reviewer for bringing this important point forward.

5 Oblique Multiple Group (OMG) analysis indicated that items measuring biospheric values and environmental self-identity correlate strongly with the respective scales. One item measuring environmental self-identity (“Acting environmentally friendly is an important part of who I am”) also correlated strongly with the biospheric values scale (*r* = 0.55), as well as with the environmental self-identity scale (*r* = 0.54); please see OSF repository https://osf.io/qn9fp/?view_only=055ffd0809454c0ba6bd2f2597097c08). Future research is needed to find out whether this strong positive correlation can be replicated and if so what causes this strong positive correlation in Lithuania; previous studies in other countries showed that all items measuring biospheric values and environmental self-identity could be empirically distinguished (van der Werff et al., 2013b, 2014b).

6 We controlled for the possible differences in the tested model across the data collected in two different ways: online versus paper questionnaire. The invariance analysis with Mplus 7.4 (Muthén and Muthén, (1998–2010)) was performed. We found no significant differences in the model fit (cf. Milfont and Fischer, 2010). We additionally tested gender effects on the studied model of relationship and found no significant differences in the tested model based on gender.

7 The value of TLI was slightly below the suggested threshold (0.95; Schreiber et al., 2006), yet it is still acceptable and does not deviate much from the acceptable values (Hooper et al., 2008; Little, 2013).
